# Supplementary material for: Comparison and benchmark of deep learning methods for non-coding RNA classification
Source: PLoS Comput Biol. 2024 Sep 12;20(9):e1012446. doi: 10.1371/journal.pcbi.1012446 (PMC11421803; doi:10.1371/journal.pcbi.1012446)
Supplement: S1 Table — (PDF) [file pcbi.1012446.s001.pdf]

Label	Dataset1<br>Avg. length	 Train size	 Test size	Dataset2<br>Avg. length	 Train size	 Test size
**5S rRNA**	119.1	500 (11)	197 (5)	119.9	3,496 (0)	1,500 (0)
**5.8S rRNA**	153.4	500 (58)	184 (21)	150.7	322 (0)	126 (0)
**CD-box**	106.0	500 (4)	195 (4)	108.3	3,492 (0)	1,504 (0)
**HACA-box**	139.8	500 (2)	194 (1)	144.7	3,514 (0)	1,485 (0)
**Intron gpI**	342.5	500 (46)	174 (11)	284.5	903 (0)	390 (0)
**Intron gpII**	95.6	500 (12)	160 (5)	139.8	2,475 (0)	1,089 (0)
**IRES**	233.5	320 (8)	124 (11)	-	-	-
**leader**	124.7	500 (3)	145 (1)	212.9	3,481 (0)	1,514 (0)
**miRNA**	108.5	500 (4)	196 (2)	116.4	3,529 (0)	1,466 (0)
**riboswitch**	142.1	500 (2)	193 (0)	142.2	3,512 (0)	1,483 (0)
**ribozyme**	259.8	500 (4)	188 (2)	303.1	3,218 (0)	1,408 (0)
**scaRNA**	174.2	500 (2)	103 (1)	-	-	-
**tRNA**	77.7	500 (3)	200 (0)	81.4	3,463 (0)	1,533 (0)
**Y RNA**	-	-	-	104.7	320 (0)	107 (0)
**Y RNA-like**	-	-	-	129.3	76 (0)	41 (0)
**ALL**	157.3	6,320 (159)	2,253 (64)	154.5	31,801 (0)	13,646 (0)

Table 1. Description of ncRNA classification datasets. Dataset1: a version of the dataset in Fiannaca et al. in which we removed the data leakage bias. **Dataset2**: the dataset by Lima et al. We present the average length of sequences from each class, as well as the number of ncRNAs in the training and test sets. In parenthesis is indicated the number of sequences containing degenerate nucleotides (i.e., other than A,C,G,T/U). These are removed from Dataset1 to form Dataset1-nd.
