# Supplementary material for: Comparison and benchmark of deep learning methods for non-coding RNA classification
Source: PLoS Comput Biol. 2024 Sep 12;20(9):e1012446. doi: 10.1371/journal.pcbi.1012446 (PMC11421803; doi:10.1371/journal.pcbi.1012446)
Supplement: S2 Table — (PDF) [file pcbi.1012446.s002.pdf]

	Method	Accuracy	MCC	F1-score	Recall	Precision	Specificity
**Dataset1**	**nRC**	0.707 ± 0.024	0.683 ± 0.026	0.698 ± 0.026	0.7 ± 0.025	0.7 ± 0.026	0.976 ± 0.002
	**RNAGCN**	0.862 ± 0.011	0.85 ± 0.012	0.857 ± 0.011	0.857 ± 0.011	0.861 ± 0.012	0.988 ± 0.001
	**ncrna-deep**	0.921 ± 0.01	0.914 ± 0.011	0.919 ± 0.01	0.919 ± 0.011	0.922 ± 0.009	0.993 ± 0.001
**Dataset1-nd**	**nRC**	0.698 ± 0.015	0.673 ± 0.017	0.692 ± 0.016	0.694 ± 0.016	0.694 ± 0.015	0.975 ± 0.001
	**RNAGCN**	0.851 ± 0.01	0.839 ± 0.01	0.848 ± 0.01	0.847 ± 0.01	0.854 ± 0.011	0.988 ± 0.001
	**ncrna-deep**	0.914 ± 0.013	0.908 ± 0.014	0.913 ± 0.014	0.912 ± 0.014	0.918 ± 0.012	0.993 ± 0.001
	**MFPred**	0.873 ± 0.021	0.863 ± 0.022	0.872 ± 0.021	0.871 ± 0.021	0.879 ± 0.02	0.989 ± 0.002
**Dataset2**	**nRC**	0.772 ± 0.007	0.746 ± 0.008	0.757 ± 0.018	0.742 ± 0.024	0.785 ± 0.012	0.98 ± 0.001
	**RNAGCN**	0.945 ± 0.004	0.939 ± 0.004	0.94 ± 0.007	0.94 ± 0.012	0.942 ± 0.009	0.995 ± 0.0
	**ncrna-deep**	0.97 ± 0.004	0.967 ± 0.004	0.974 ± 0.004	0.971 ± 0.007	0.978 ± 0.002	0.997 ± 0.0
	**MFPred**	0.954 ± 0.012	0.949 ± 0.013	0.958 ± 0.01	0.955 ± 0.009	0.964 ± 0.011	0.996 ± 0.001

Table 2. 10-fold cross-validation mean and standard deviation of different metrics.
