# Supplementary material for: Comparison and benchmark of deep learning methods for non-coding RNA classification
Source: PLoS Comput Biol. 2024 Sep 12;20(9):e1012446. doi: 10.1371/journal.pcbi.1012446 (PMC11421803; doi:10.1371/journal.pcbi.1012446)
Supplement: S3 Table — (PDF) [file pcbi.1012446.s003.pdf]

	Method	Accuracy	MCC	F1-score	Recall	Precision	Specificity
**Dataset1**	**nRC**	0.746	0.725	0.743	0.753	0.738	0.979
	**RNAGCN**	0.865	0.854	0.861	0.864	0.863	0.989
	**ncrna-deep**	0.944	0.939	0.944	0.943	0.946	0.995
	**ncRDense**	0.913	0.906	0.912	0.91	0.92	0.993
**Dataset1-nd**	**nRC**	0.75	0.729	0.748	0.756	0.747	0.979
	**RNAGCN**	0.872	0.861	0.868	0.871	0.871	0.989
	**ncrna-deep**	0.95	0.946	0.95	0.951	0.951	0.996
	**MFPred**	0.907	0.899	0.911	0.909	0.916	0.992
	**ncRDense**	0.912	0.905	0.912	0.91	0.92	0.993
	**NCYPred**	0.798	0.783	0.716	0.744	0.705	0.983
**Dataset2**	**nRC**	0.783	0.758	0.774	0.76	0.793	0.981
	**RNAGCN**	0.947	0.941	0.944	0.936	0.954	0.995
	**ncrna-deep**	0.971	0.968	0.97	0.965	0.976	0.998
	**MFPred**	0.965	0.961	0.968	0.962	0.974	0.997
	**ncRDense**	0.735	0.712	0.52	0.562	0.549	0.981
	**NCYPred**	0.916	0.906	0.909	0.895	0.926	0.993

Table 3. Evaluation of different metrics on held-out test sets.
