# Supplementary material for: Comparison and benchmark of deep learning methods for non-coding RNA classification
Source: PLoS Comput Biol. 2024 Sep 12;20(9):e1012446. doi: 10.1371/journal.pcbi.1012446 (PMC11421803; doi:10.1371/journal.pcbi.1012446)
Supplement: S4 Table — (PDF) [file pcbi.1012446.s004.pdf]

		Computation time			Emissions
		**Preprocessing**	**Training**	**Prediction**	(in gCO_2eq)
**Dataset1**	**nRC**	23mn 54s	2h	12s	-
	**RNAGCN**	2mn52	17mn 17s	$< 1s$	5.9
	**ncrna-deep**	2s	59s	$< 1s$	0.3
**Dataset2**	**nRC**	3h 7mn	9h 4mn	57s	-
	**RNAGCN**	13mn 22s	1h 5mn	2s	17.8
	**ncrna-deep**	11s	4mn 11s	2s	1.7
	**MFPred**	2mn 53s	9h 19mn	1mn 3s	233.8

Table 4. Comparison of computation times and CO_2 emissions on Dataset1 and Dataset2. The computation time is calculated for preprocessing, training and prediction, while the CO_2 emission is calculated for training.
