# Supplementary material for: Comparison and benchmark of deep learning methods for non-coding RNA classification
Source: PLoS Comput Biol. 2024 Sep 12;20(9):e1012446. doi: 10.1371/journal.pcbi.1012446 (PMC11421803; doi:10.1371/journal.pcbi.1012446)
Supplement: S1 Fig — (PDF) [file pcbi.1012446.s005.pdf]

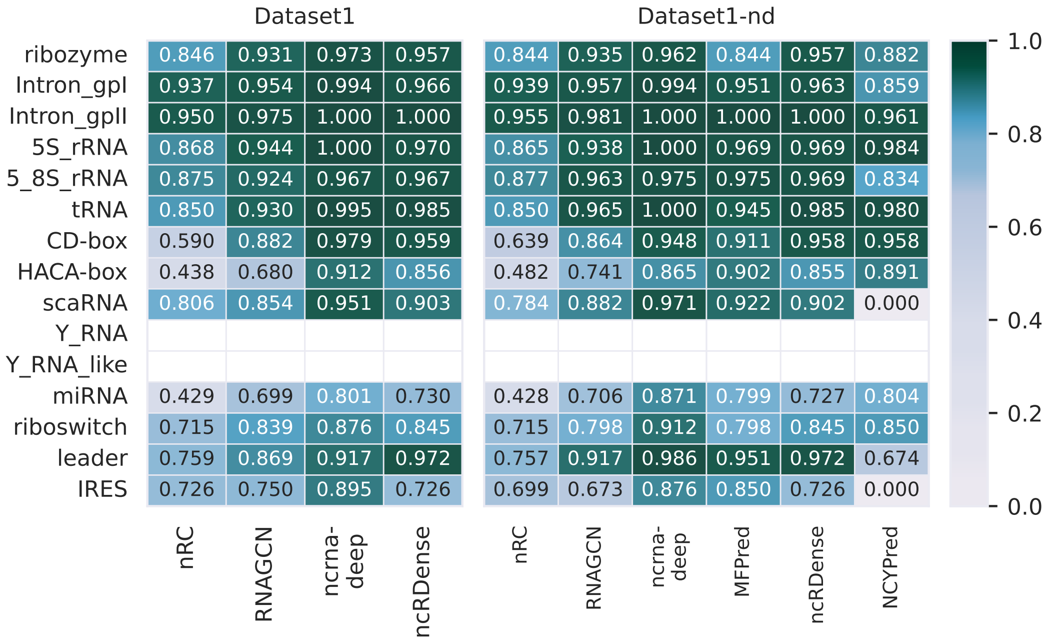

Figure 1. Comparison of accuracy of prediction of each ncRNA class obtained by state-of-the-art tools on Dataset1 and Dataset1-nd. Light colors correspond to lower accuracies, while colors tending towards dark green represent the best results. Note that results cannot be obtained on Dataset1 for MFPred and NCYPred as these methods cannot predict sequences containing degenerate nucleotides.
