# Supplementary figures and images for: Comparison and benchmark of deep learning methods for non-coding RNA classification
Source: PLoS Comput Biol. 2024 Sep 12;20(9):e1012446. doi: 10.1371/journal.pcbi.1012446 (PMC11421803; doi:10.1371/journal.pcbi.1012446)

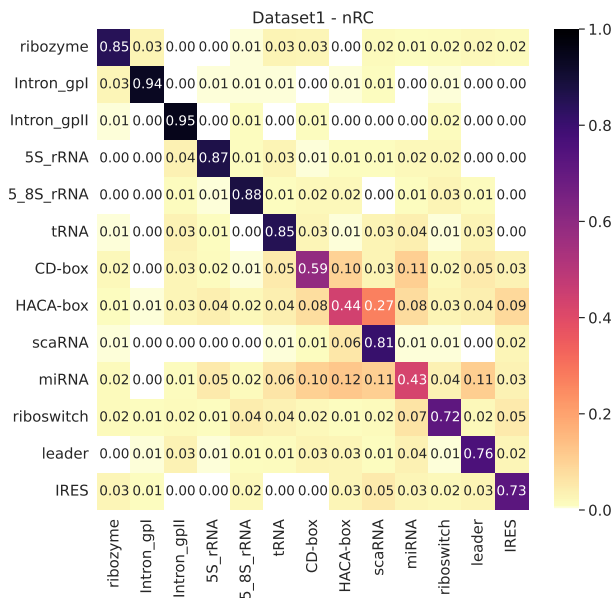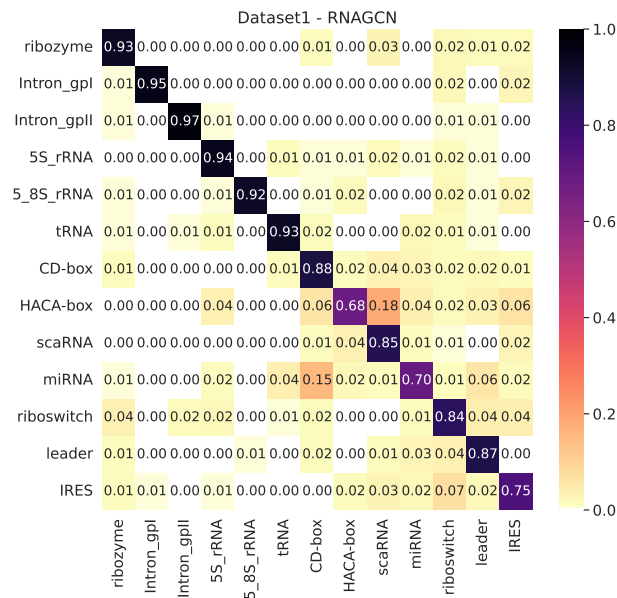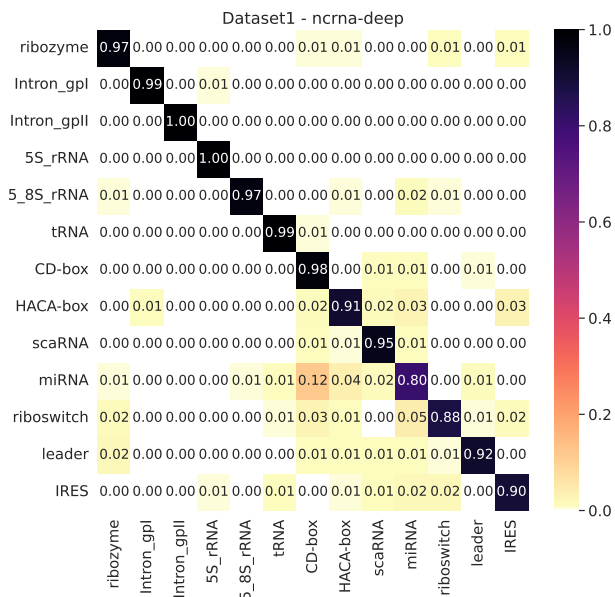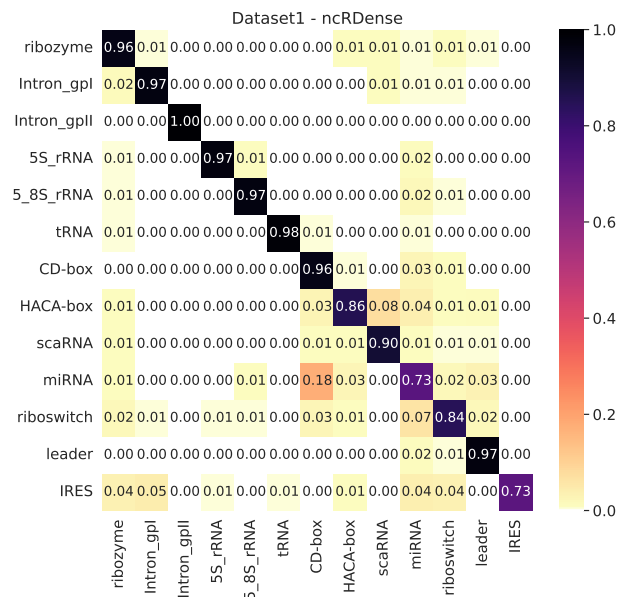

Figure 2. Confusion matrices on Dataset1.

Supplement: S2 Fig — (PDF) [file pcbi.1012446.s006.pdf]

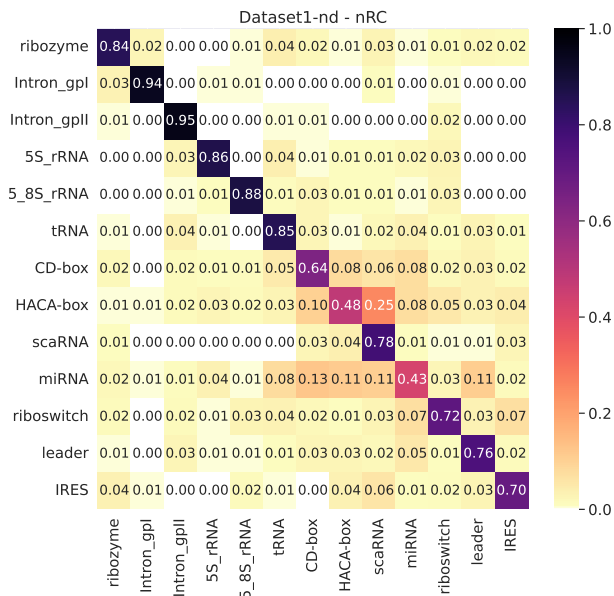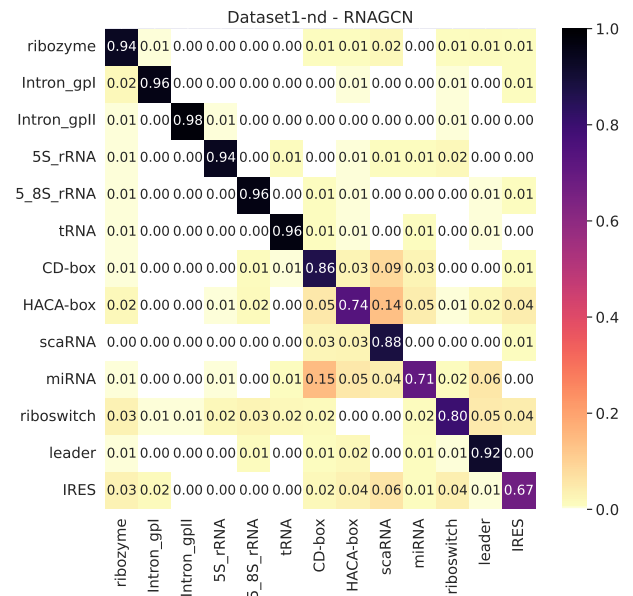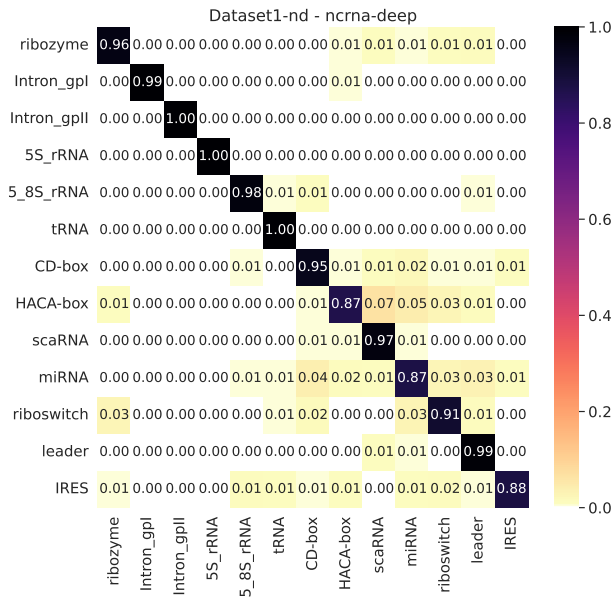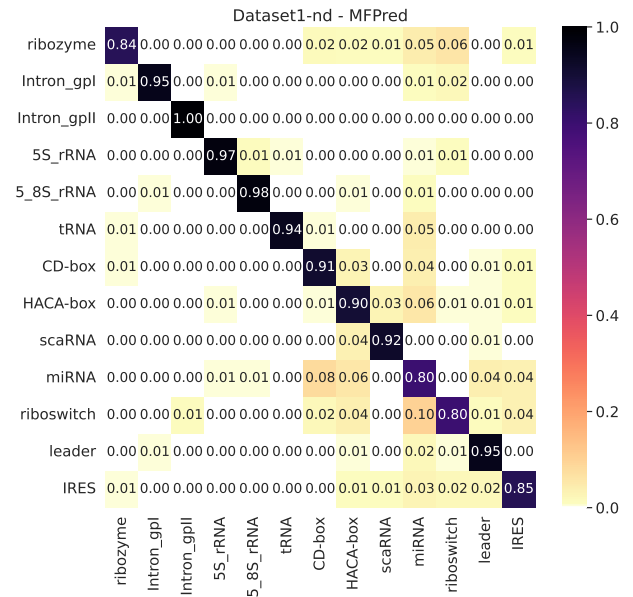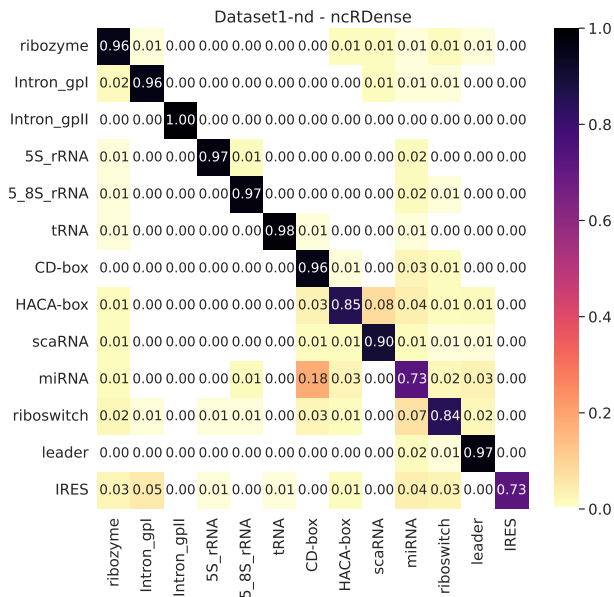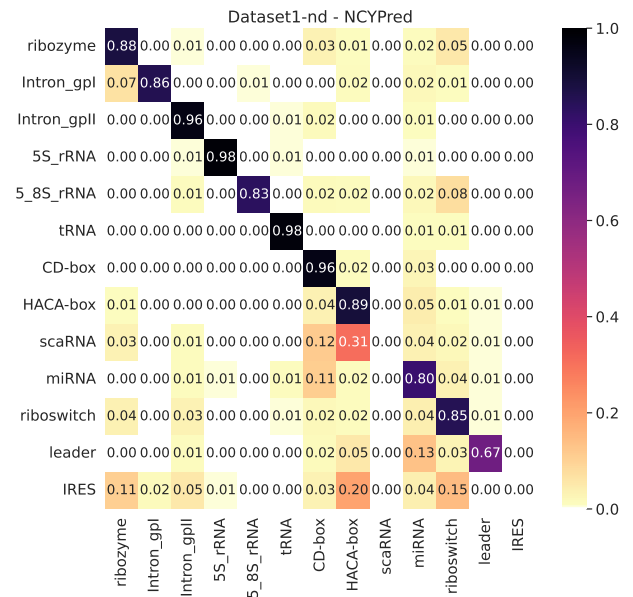

Figure 3. Confusion matrices on Dataset1-nd.

Supplement: S3 Fig — (PDF) [file pcbi.1012446.s007.pdf]

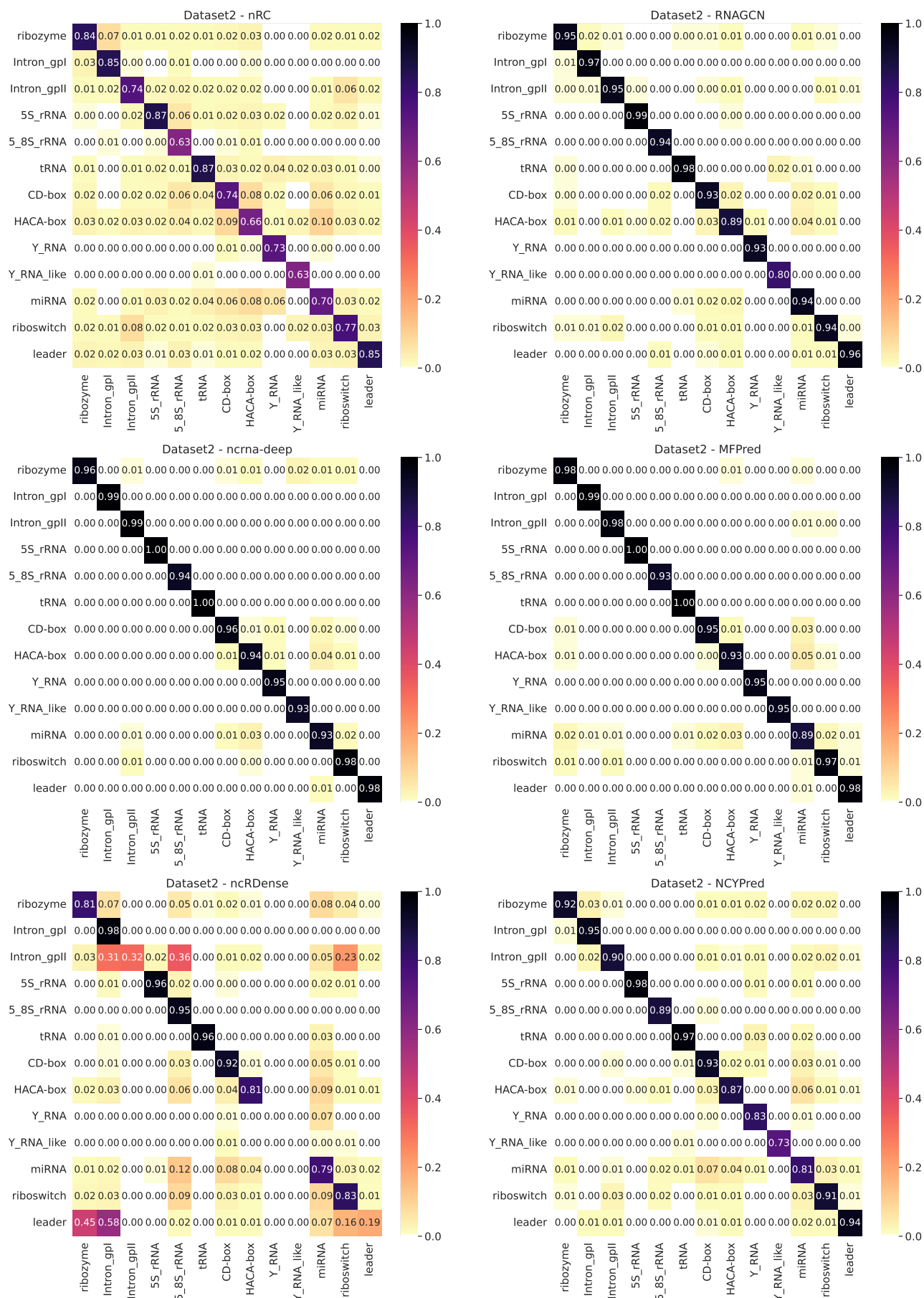

**Figure 4. Confusion matrices on Dataset2.**

Supplement: S4 Fig — (PDF) [file pcbi.1012446.s008.pdf]
